# Far eastern curlew and whimbrel prefer flying low - wind support and good visibility appear only secondary factors in determining migratory flight altitude

**DOI:** 10.1186/s40462-021-00267-5

**Published:** 2021-06-13

**Authors:** Batbayar Galtbalt, Amanda Lilleyman, Jonathan T. Coleman, Chuyu Cheng, Zhijun Ma, Danny I. Rogers, Bradley K. Woodworth, Richard A. Fuller, Stephen T. Garnett, Marcel Klaassen

**Affiliations:** 1 Centre for Integrative Ecology, School of Life and Environmental Science, Deakin University, Geelong, Victoria Australia; 2 Threatened Species Recovery Hub, National Environment Science Program, Research Institute for Environment and Livelihoods, Charles Darwin University, Ellengowan Drive, Casuarina, Northern Territory 0909 Australia; 3 Queensland Wader Study Group, 22 Parker Street, Shailer Park, 4128 Australia; 4 Ministry of Education Key Laboratory for Biodiversity Science and Ecological Engineering, Coastal Ecosystems Research Station of the Yangtze River Estuary, Institute of Biodiversity Science, School of Life Sciences, Fudan University, Shanghai, 200433 China; 5 Department of Environment, Water, Land and Planning, Arthur Rylah Institute, PO Box 137, Heidelberg, Victoria 3084 Australia; 6 Australasian Wader Studies Group, Melbourne, Victoria Australia; 7 School of Biological Sciences, University of Queensland, Brisbane, Queensland Australia; 8 Victorian Wader Study Group, Melbourne, Victoria Australia

**Keywords:** Air temperature, Altitude selection, Shorebird, Atmospheric condition, Weather, Migration

## Abstract

**Background:**

In-flight conditions are hypothesized to influence the timing and success of long-distance migration. Wind assistance and thermal uplift are thought to reduce the energetic costs of flight, humidity, air pressure and temperature may affect the migrants’ water balance, and clouds may impede navigation. Recent advances in animal-borne long-distance tracking enable evaluating the importance of these factors in determining animals’ flight altitude.

**Methods:**

Here we determine the effects of wind, humidity, temperature, cloud cover, and altitude (as proxy for climbing costs and air pressure) on flight altitude selection of two long-distance migratory shorebirds, far eastern curlew (*Numenius madagascariensis*) and whimbrel (*Numenius phaeopus*). To reveal the predominant drivers of flight altitude selection during migration we compared the atmospheric conditions at the altitude the birds were found flying with conditions elsewhere in the air column using conditional logistic mixed effect models.

**Results:**

Our results demonstrate that despite occasional high-altitude migrations (up to 5550 m above ground level), our study species typically forego flying at high altitudes, limiting climbing costs and potentially alleviating water loss and facilitating navigation. While mainly preferring migrating at low altitude, notably in combination with low air temperature, the birds also preferred flying with wind support to likely reduce flight costs. They avoided clouds, perhaps to help navigation or to reduce the risks from adverse weather.

**Conclusions:**

We conclude that the primary determinant of avian migrant’s flight altitude selection is a preference for low altitude, with wind support as an important secondary factor. Our approach and findings can assist in predicting climate change effects on migration and in mitigating bird strikes with air traffic, wind farms, power lines, and other human-made structures.

**Supplementary Information:**

The online version contains supplementary material available at 10.1186/s40462-021-00267-5.

## Background

The air through which birds, bats, and many insects travel may vary considerably in temperature, humidity, air pressure, visibility, wind speed and wind direction depending on the altitude at which these animals travel. These factors affect the costs and the risks associated with long-distance travel, such as during migration [1–4]. Wind conditions and how these vary with altitude have most frequently been considered the primary factor affecting migratory flight [2, 5–10]. Similarly, thermal uplift is crucial for soaring migrants, its intensity determining the altitudes and subsequent gliding distance that birds can attain (9, references therein). The roles of humidity, temperature and pressure have also been considered, notably in relation to their potential impacts on migrants’ water balance [5, 11]. Finally, poor visibility (cloud cover) and precipitation may increase the costs and risks of migration and have indeed been found to reduce migratory onset and intensity [9] and references therein, [12, 13]. Given this range of potential effects of atmospheric variables on the energy and water balance of avian migrants and, thus, the costs and success of migration, decisions on when to fly and at what altitude are probably of paramount importance to aerial migrants.

Most studies on flight altitude selection in migratory birds have been based on radar observations and suggest that migratory flights typically occur up to 1500 m above ground level (a.g.l.), although much higher altitudes are also occasionally recorded [1, 2, 9, 14]. Wind, which may provide assistance to migrating birds, can vary greatly in speed and direction with altitude [15] and studies in the trade wind zone (i.e. between 30°N and 30°S) have found that the flight altitude of birds at these latitudes coincides with profitable winds, suggesting migrants choose altitudes that provide the best wind support [6, 7, 16]. However, in the mid-latitudes (i.e. 30°N - 60°N, and 30°S - 60°S), studies have suggested that migrants may not always fly at the altitude that provides the most favourable winds [17, 18]. A radar study in southern Italy suggested that diurnal migrants were not always selecting the altitude with maximum tailwind, instead climbing until wind support no longer improved, irrespective of even better wind support at higher altitudes [18]. Similarly, using weather radar observations in the Netherlands, Kemp et al. [17] found that nocturnal migratory birds mainly flew at low altitudes unless there were headwinds, also suggesting birds do not always select the altitude with the best wind support.

Atmospheric factors other than wind and thermals have rarely been investigated in the context of migrants’ flight altitude selection. High temperature, low humidity and low air pressure have been suggested to cause water loss due to evaporative cooling, limiting migration distances and flight durations [5, 11]. However, radar studies addressing this have found mixed or limited support, often because ideal conditions for optimal flight conditions from an energetic and water balance perspective overlapped [19, 20]. Regarding the impact of visibility and thus clouds on flight altitude selection, birds have been found to fly both above, below and even in the clouds [9] and references therein, [21]. However, systematic tests of the effect of clouds on altitude selection are few. In one study, Kemp et al. [17] found no effect of cloud cover on altitude selection of nocturnal migrants over the Netherlands, although their study may have been biased by selecting nights with intense migration only. While Kahlert et al. [22] found that waterbirds flew low under cloud cover, they acknowledged that flights above the clouds might have remained undetected in their study [22]. Thus, the role of cloud cover in selecting migration flight altitude remains largely unresolved.

There are several limitations with radar studies when studying animals’ flight altitude during migration. Firstly, radars capture only a small fraction of a migratory journey, which may not be representative of the whole migratory episode, notably when they are potentially recording the initial (ascent) or final (descent) sections of a migratory leg. Secondly, migrants flying close to the ground or below the radar horizon cannot be detected [1, 2] while also very high altitude migrations have an increased chance of being missed. Furthermore, detailed information on flight altitude over sea, far away from land, can rarely be detected using radar observations (but see [23].

As an alternative to radar studies, a few animal-borne tracking studies have also investigated migrants’ flight altitude, often highlighting the apparent importance of wind support. For example, Bewick’s swan (*Cygnus columbianus bewickii*) migrating from Denmark to Northern Russia flew at low altitudes with some tailwind support, even though more favourable tailwinds were available at higher altitudes [24]. More recently, a tracking study of four individual black-tailed godwits (*Limosa limosa*) migrating from The Netherlands to sub-Saharan Africa suggested that high flight altitudes were associated with high air temperature at low altitudes and increasing wind support at higher altitudes [25]. Similarly, Eurasian curlews (*Numenius arquata*) selected higher altitudes if there were headwinds at lower altitudes when departing from the Wadden Sea during northbound migration [26]. Despite these findings, knowledge of determinants of flight altitude in migratory birds from individual tracking remains limited. In a recent paper describing the contrast between night-time (mean 2394 m above sea level, a.s.l.) and daytime (5367 m a.s.l.) flight altitude in migrating great reed warblers (*Acrocephalus arundinaceus*) crossing the Mediterranean Sea and Sahara Desert, a call was made to study diel variation in ambient temperature, wind, predation, vision range, and solar radiation as potential drivers for this spectacular phenomenon [14].

Thus, despite a considerable body of literature on flight altitude and the perceived importance of conditions aloft on migration strategies and the success of migratory journeys, there is limited quantitative understanding of where in the air column migratory birds fly during their journeys and how that correlates with conditions aloft. More specifically, under the assumption that migrants select flight altitudes with the aim to minimise the duration and metabolic costs of migration, there is a need to test the following hypotheses and evaluate their importance: (i) migrants fly at altitudes that maximise wind support, (ii) migrants maximise visibility (i.e. no or fewer clouds), (iii) migrants fly at altitudes that reduce the chance of overheating and minimise water loss, and (iv) migrants avoid climbing costs by flying at lower altitudes. Addressing these hypotheses will improve our understanding of migration strategies and the physiological and ecological challenges that migrants face. Moreover, since atmospheric conditions are predicted to shift with a changing climate, understanding flight altitude selection may also allow predictions on how climate change affects migratory flights, a major question that has not yet been considered. Finally, being able to predict altitudes of migratory flights is important for air traffic control and mitigating bird strikes.

To test the above hypotheses, we determined the relative importance of wind support, temperature, humidity, cloud cover, and altitude (as a proxy for climbing costs) for in-flight altitude selection of two long distance migrants, far eastern curlew (*Numenius madagascariensis*) and whimbrel (*Numenius phaeopus*). Both species migrate between their Australian non-breeding grounds and their breeding grounds in northern China and eastern Siberia along the East Asian-Australasian Flyway. To this end, we modelled the atmospheric conditions in the air column at every in-flight fix by comparing the condition at the actual flight altitude with conditions elsewhere in the air column using conditional logistic mixed effect models.

## Methods

### Tracking data

**Fig. 1:**
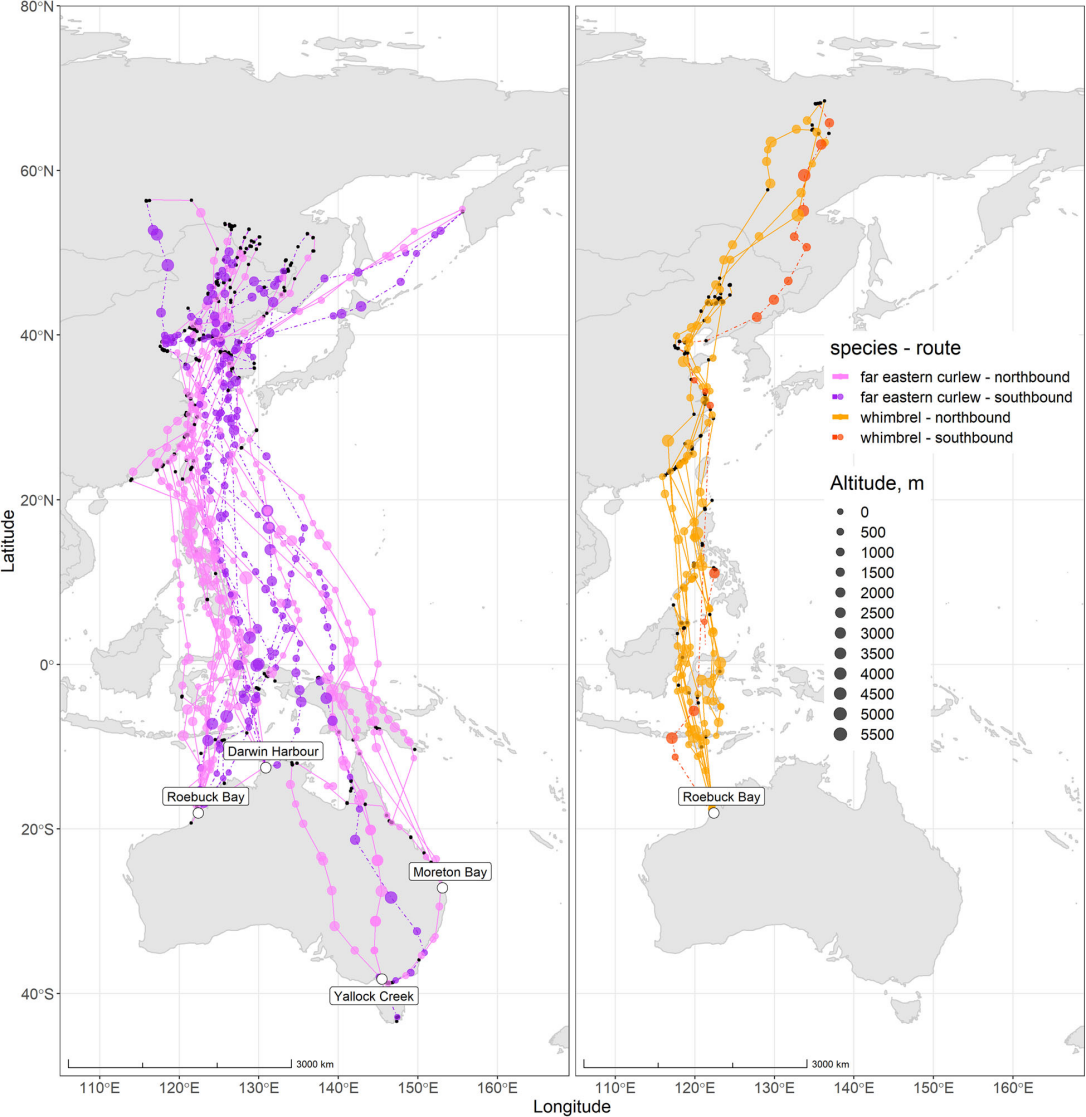
Migratory routes of far eastern curlew (purple) and whimbrel (brown). Sites of transmitter deployment are marked by white dots. In-flight fixes are marked by coloured dots where size indicates the flight altitude. Stationary sites, including stopover sites, are marked by black dots

We used tracking data from 17 far eastern curlews and 9 whimbrels. Birds were caught using cannon netting or mist netting by the Australasian Wader Studies Group, the Queensland Wader Study Group and the Victorian Wader Study Group at various non-breeding sites around Australia in 2017 and 2018 (details provided in additional file Table S1; Fig. 1). GPS-Global System for Mobile Communication (GSM) transmitters (Ornitela for far eastern curlew; Hunan Global Messenger Technology, China for whimbrel) were deployed on the back of each individual and secured using a leg-loop harness [27]. The weight of the transmitters was 15 g or 20 g for far eastern curlew (1–3% of body mass (Australian males 696.6 ± 52.86 g and females 796.7 ± 54.26 g in October after migration; males 1089.3 ± 73.18 g and females 1224.5 ± 69.27 g in March shortly before migration [28])) and 7 g for whimbrel (~ 2% of body mass (405.1 ± 28.9 g upon deployment)). All tagged birds were aged as adults on capture on the basis of plumage and moult characteristics [28]. Transmitters of far eastern curlew were programmed to take a position at 6 h intervals while those of whimbrel were programmed to take positions at 2 and 6 h intervals. For both transmitter types the nominal geographic positioning accuracy was ±10 m.

The tracking data of the 17 far eastern curlews mentioned above comprised 19 northbound and 11 southbound migratory tracks with a total of 27,021 fixes, while for the 9 whimbrels we had 10 northbound and 1 southbound migratory track with a total of 2114 fixes. Many transmitters failed after northbound migration, resulting in smaller samples for southward migration. None of the transmitters stopped functioning while birds were actively migrating, and in those transmitters that failed, the tracks received shortly before the transmitters ceased functioning gave us no reason to suspect birds were behaving abnormally. We assume the transmitters had no impact on the birds’ flight behaviour. Based on the distribution of instantaneous measurements of ground speed, we classified the point fixes as either in-flight or stationary, using a ground speed of 7.5 m/s as a cut-off (additional file Fig. S1). In addition, we retained only those in-flight fixes with a step length (i.e. distance between the current and the next fix) larger than 30 km (assuming a minimum flight speed of 7.5 m/s = 27 km/h), to make it likely that in-flight fixes are representing migratory flights rather than local movements. Although the nominal altitudinal accuracy of the trackers was ±30 m the realised accuracy was poorer as evidenced by 7% of the in-flight fixes in far eastern curlew having a negative altitude estimate. Five percent of these 7% were 30 m below the surface and 6% were 80 m below the surface, with the most extreme estimate being 527 m below the surface. Imprecision and negative altitudinal measurements are a common issue in tracking studies [29] and often it is recommended to smooth altitudinal data using state space modelling [30]. However, we were not able to smooth the data using such modelling because the interval between fixes was larger than the recommended threshold of one hour. In principle, the higher than nominal error in altitudinal measurements will reduce the amount of explained variance in our altitude selection analysis, but not bias the results, provided there is also no bias in the altitudinal measurement error. Moreover, in the conditional logistic mixed effect modelling that we used to evaluate what factors determine the birds’ selection of altitudinal air layer we used 13, relatively wide (500 m) altitudinal air layers (for details see below). Finally, in order to avoid causing bias in the dataset, we kept negative altitude in-flight fixes, assuming they represent near-surface in-flight fixes (i.e. 10 m a.g.l.) [30].

Ultimately, we retained 512 migratory in-flight fixes for far eastern curlew and 146 migratory in-flight fixes for whimbrel [31]. We classified all in-flight fixes as either flights over land or over sea and during either day or night. Whether in-flight fixes took place over land or over sea was identified using the “over” function in R package sp. [32]. To identify day and night fixes, we made use of the times of sunrise and sunset for each in-flight fix on their corresponding day and location which were calculated using the R package suncalc [33].

### Aloft variables (wind, cloud cover, temperature and relative humidity)

For each in-flight fix the land elevation and the wind condition, cloud cover, air temperature, and relative humidity near the surface and at every 500 m up to 6000 m above mean sea level was obtained. These environmental variables were downloaded from the European Centre for Medium-range Weather Forecast’s (ECMWF) global atmospheric re-analysis dataset [34] using the Env-DATA annotation system within the animal movement data portal Movebank [35]. The spatial accuracy of this data source was 0.75 degrees and the temporal accuracy was six hours. Hence, to obtain most probable estimates for the environmental variables we used bilinear spatio-temporal interpolation available within the Env-DATA system. Wind data is provided in west to east (zonal, V_z_, m/s) and south to north (meridional, V_m_, m/s) wind components. We used these wind components together with flight direction (i.e. the direction in which the bird flies, θ_f,_ which was estimated based on consecutive fixes using the ‘angle’ function within package move [36]) to calculate wind support (WS, m/s) following Safi et al. [37] using:

$$ WS=\sqrt{{V_m}^2+{V_z}^2}\times \cos \left(\mathrm{atan}2\left({V}_m,{V}_z\right)-\frac{2{\pi \theta}_f}{360}\right) $$

The Env-DATA annotation system does not provide near-surface cloud cover data. Hence, we used the cloud cover for the lowest altitude for which cloud cover data is available (i.e. 200 m a.s.l.) instead. Near surface relative humidity was calculated from air temperature and dew-point temperature, which were also annotated via the EnvDATA system, using Clausius-Clapeyron approximation [38].

### Statistical analysis

For each species separately, we used conditional logistic mixed effect modelling to evaluate the contribution of the conditions aloft on the birds’ selection of flight altitude at each in-flight GPS fix. To this end, we constructed 13 altitudinal layers ranging from 0 to 250 m a.s.l., 250–750 m a.s.l., 750–1250 m a.s.l., etc. up to 6250 m a.s.l. For each in-flight fix, the EnvDATA sourced environmental variables were assigned to their respective altitudinal layer and the layer in which the bird was found to fly was marked as a ‘case’ layer, and the remaining 12 layers as ‘control’ layers. Thus, the sampling units in our analyses are the air columns of each in-flight fix, each of which consists of one case (i.e. selected layer = 1) and 12 control layers (i.e. unselected layers = 0). The cases and controls collectively form the response variable and the conditional logistic mixed effect model calculates which explanatory variables significantly explain the birds’ selection among air layers. As explanatory variables we used wind support, cloud cover, temperature, and relative humidity in each of the 13 alternative altitudinal layers. The centre value for altitude in each layer was also included as an explanatory variable in the model as a proxy for climbing costs. This should not be regarded as a circular function, i.e. flight altitude being used to estimate flight altitude. Rather, we investigated whether flight altitude was one of the variables which affected the air layer that the birds chose to fly in. We additionally used individuals as random intercept in order to account for repeated measures and different numbers of fixes per individual. We used the coxme package [39] to conduct the conditional logistic mixed effect model in R version 4.0.2 [40]. All explanatory variables were centred and z-transformed, to enable comparison of coefficients for effect size.

To further describe the migrants’ flight behaviour in relation to cloud cover (i.e. if they avoid clouds by flying under or over it), we calculated the percentage of in-flight fixes above the clouds, below the clouds, in the clouds and under clear conditions (i.e. no clouds in the air column). We considered “cloudy” to be conditions with cloud cover values exceeding 70%.

## Results

**Fig. 2:**
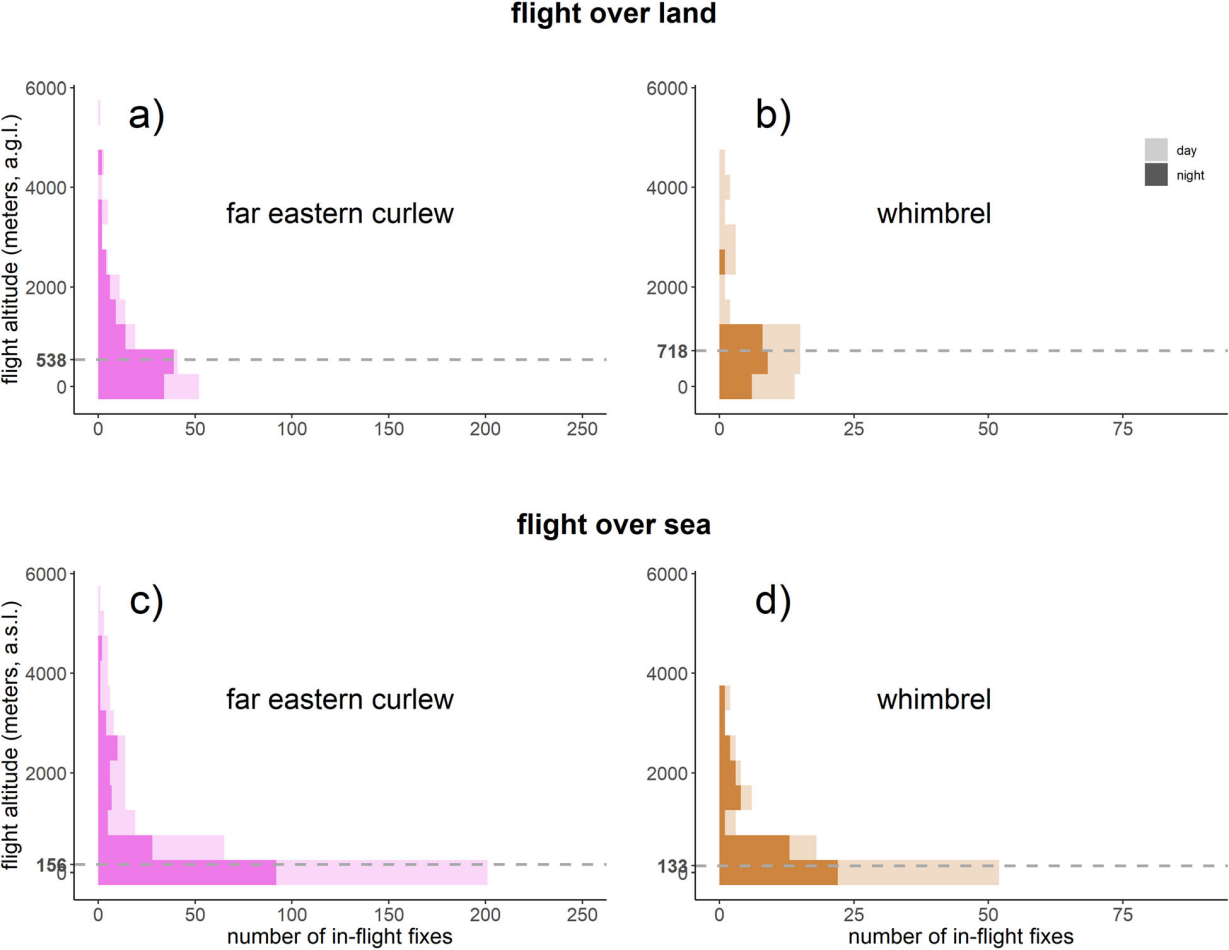
Histograms depicting the vertical distribution of flight altitudes of far eastern curlew and whimbrel over land (top panels) and sea (bottom panels) during both day (light shading) and night (dark shading). Dashed lines indicate median altitudes

Both species mainly flew at altitudes below 1000 m a.g.l. (i.e. 75% of the time in both far eastern curlew and whimbrels; Fig. 2). However, they occasionally flew higher, far eastern curlew reaching altitudes as high as 5550 m a.g.l., and whimbrels as high as 4471 m a.g.l. There was no difference in flight altitude between the two species (Wilcoxon test, w = 34,861, *p* = 0.21).

Far eastern curlew tended to fly higher over land (median = 538 m a.g.l.; *n* = 155) than over sea (median = 156 m a.s.l.; *n* = 357) (Wilcoxon test, w = 15,960, *p* < 0.001). Flight altitudes of far eastern curlew did not differ between day and night flights, whether it was over sea (Wilcoxon test, w = 16,715, *p* = 0.30) or over land (Wilcoxon test, w = 2551, *p* = 0.57, Fig. 2).

Whimbrel also flew higher over land (median: 718 m a.g.l.; *n* = 57) than over sea (median: 133 m a.s.l.; *n* = 89; Wilcoxon test, w = 3607, p < 0.001). The flight altitudes did not differ between day and night flights over sea (Wilcoxon test, w = 792, *p* = 0.11), but over land they flew higher during daytime than nighttime (median: 1057 m a.g.l.; *n* = 33 and 412 m a.g.l.; *n* = 24, for day and night, respectively; Wilcoxon test, w = 527, *p* < 0.05, Fig. 2).

The conditions at the actual migratory flight altitude (± 250 m) of far eastern curlews and whimbrels were only slightly different (see details in Table S2 in additional file). Most of the explanatory, in-flight variables were correlated with each other to varying extents, except for wind support (additional file Fig. S2). Unsurprisingly there was a strong negative correlation between air temperature and altitude. To account for potential collinearity between these two explanatory variables, we included the interaction between the two in the conditional logistic regression models of both species.

**Fig. 3:**
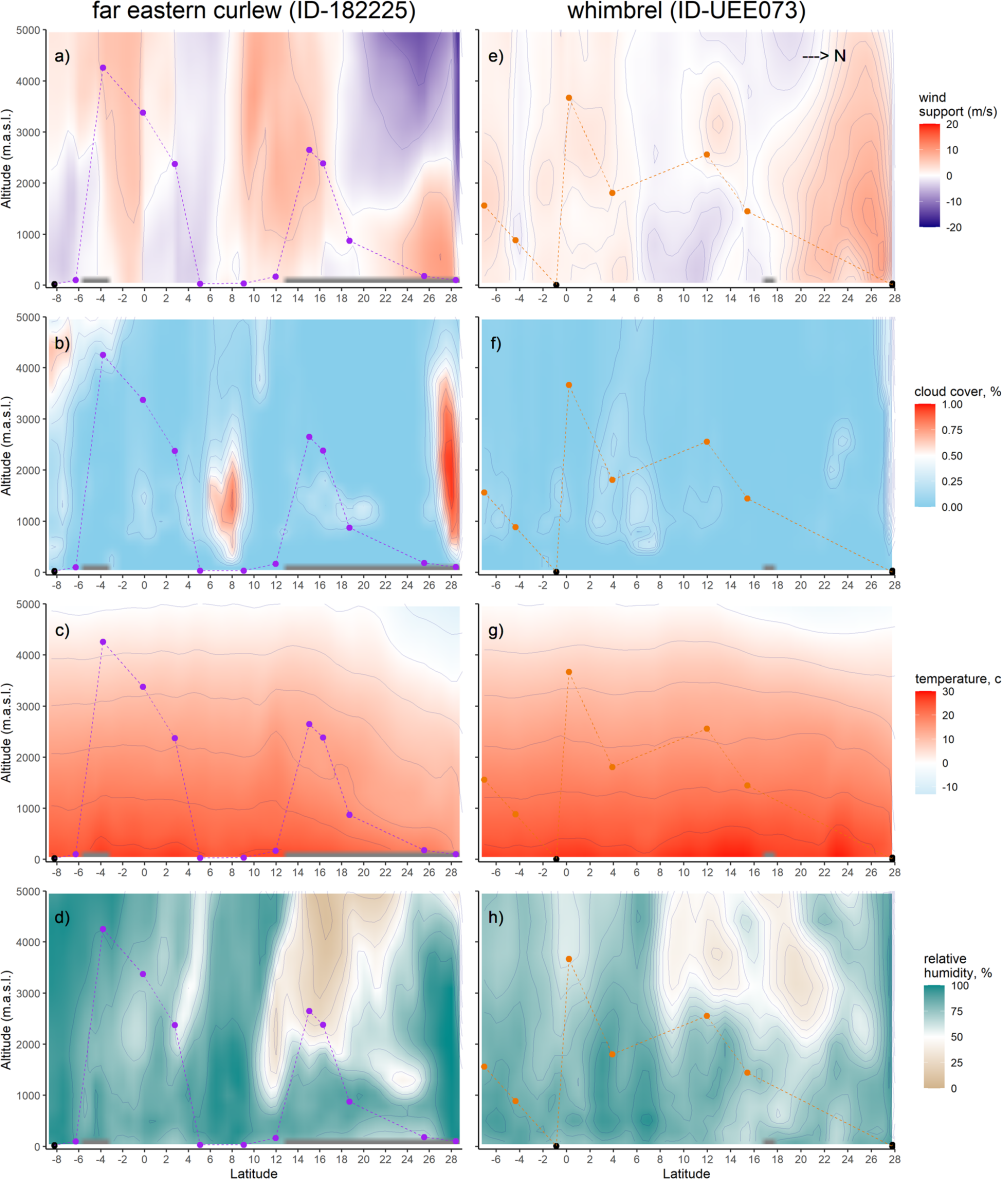
Environmental conditions through which a far eastern curlew (panel on the left; ID-182225) and whimbrel (panel on the right; ID-UEE073) migrated during a section of their northbound journey from Merauke, Indonesia to East China sea and from Kupang, Indonesia to Wenzhou, China, during the period 14–18 March 2019 and 20–25 April 2018, respectively. Coloured dots indicate individuals’ in-flight fixes while black dots indicate stationary fixes, for wind support (panels a, e), cloud cover (b, f), temperature (panels c, g) and relative humidity (panels d, h)

The conditional logistic regression analyses showed that both species had a strong preference for flying low and next had a preference for flying at altitudes where they could gain wind assistance, as well as at low altitudes with low temperatures (i.e. significant negative altitude and air temperature interaction; Table 1). Estimated effect sizes for the six explanatory variables was similar across species. Consequently, the odds ratios of the two models also highlight the similarity across both species in how altitude and conditions aloft determine the flight altitude at which the birds were recorded flying. In more detail, the odds ratios for altitude suggest that with each (scaled) unit of increase in altitude, the birds were (1–0.11 =) 89% and (1–0.08 =) 92% less likely to be flying at that altitude for far eastern curlew and whimbrel, respectively. Wind support also had a strong positive effect in determining flight altitude, its odds ratio suggesting that the chance of flying at a specific altitude increased about two-fold with every (scaled) unit of increase in wind support in both species. The interaction between altitude and air temperature was significant in both species, yet had a relatively low odds ratio and thus a small effect. Finally, albeit only in far eastern curlew, cloud cover had a slight negative effect on flight altitude (with a similar trend in whimbrel), reducing the chance of finding a bird flying at any given altitude with 32% if the cloud cover increased with one (scaled) unit. Conditions aloft during migration are depicted for two representative individuals, one of each species (Fig. 3).

**Table 1:** Effects of atmospheric conditions and altitude on far eastern curlew’s and whimbrel’s flight altitude selection as estimated using conditional logistic mixed effect modelling. All explanatory variables are scaled. Matched sample size of the model was 6127, including 482 cases, in far eastern curlew and 1832, including 146 cases, in whimbrel. Coefficients of variables, standard errors (SE), 95% confidence intervals (CIs), odds ratios and associated *p*-values of variables are presented. *P* < 0.05 are shown in bold. The interaction between altitude and air temperature is included to account for collinearity

	Coefficient (±SE)	95% CI	Odds ratio	Z	p	Random effect	
**Far eastern curlew**						σ^2^	Std. dev.
Altitude	−2.19 (0.44)	−3.05/−1.33	0.11	−4.99	**< 0.001**		
Wind support	0.83 (0.15)	0.55/1.12	2.30	5.73	**< 0.001**		
Cloud cover	−0.38 (0.10)	− 0.58/− 0.19	0.68	−3.80	**< 0.001**		
Relative humidity	0.20 (0.17)	−0.13/0.53	1.22	1.18	0.24		
Air temperature	−0.58 (0.45)	−1.45/0.30	0.56	−1.29	0.20		
Altitude: Air temperature	−0.21 (0.08)	−0.36/− 0.06	0.81	−2.69	**< 0.01**		
Individual	–	–	–	–	**–**	0.02	0.0004
**Whimbrel**							
Altitude	−2.56 (0.94)	−4.41/−0.71	0.08	−2.71	**< 0.01**		
Wind support	0.59 (0.26)	0.08/1.10	1.81	2.27	**< 0.05**		
Cloud cover	−0.04 (0.23)	−0.49/0.41	0.96	−0.17	0.86		
Relative humidity	−0.51 (0.29)	−1.09/0.06	0.60	−1.76	0.08		
Air temperature	−0.61 (0.98)	−2.53/1.30	0.54	−0.63	0.53		
Altitude: Air temperature	−0.32 (0.13)	−0.58/− 0.06	0.73	−2.45	**< 0.05**		
Individual	–	–	–	–	**–**	0.02	0.0004

Both species undertook their migratory flights mostly on clear days; 30.0% and 29.4% of the in-flight fixes for far eastern curlew and whimbrel, respectively, had no cloud present in the air column, while 77.3% and 69.7% of in-flight fixes had less than 20% of cloud cover. Only 6% (31/538) and 5% (7/150) of the in-flight fixes occurred with a cloud cover exceeding 70%. In such circumstances, 71% (22/31) of far eastern curlew fixes were below the clouds, 26% (8/31) above them and 3% (1/31) within the clouds. Whimbrel flew under the clouds in all cases (7/7).

## Discussion

Although all four initial predictions were supported by our results, the order of relative importance differed from our original expectation. Contrary to the general perception that wind support is the most important factor determining the flight altitude of migrants (e.g. [6, 8,15]), our results show that in these long distance migrants the primary determinant of flight altitude selection is a preference for flying at low altitude, which is next modulated by wind support, air temperature and visibility. Both far eastern curlew and whimbrel tended to fly at a low altitude (hypothesis iv). Next, they chose flight altitude with respect to wind support (hypothesis i). The hypothesis on visibility (ii) was also supported for far eastern curlew only, whimbrel showing a similar trend. Finally, although the birds did not appear to select altitudes with higher relative humidity and low air temperature, there was a significant effect of altitude as well as a significant interaction effect between altitude and air temperature, suggesting that birds preferred flying at low altitude and notably when those air layers were cool. Both those preferences may have assisted in reducing their water loss (hypothesis iii).

### Benefits of flight at relatively low altitudes

One of the key predictions of optimal migration theory [41] is that the energy costs for migration are minimised. From an aerodynamic perspective, high altitude migration at low air density reduces frictional resistance yet decreases lift, requiring higher flapping frequency and increasing energy costs of flight per unit air-distance covered [42]. Moreover, there is an energetic cost of climbing to higher altitude [42, 43]. Thus, not considering the potential for wind assistance, remaining at low altitude could importantly contribute to minimizing energetic costs in migrants. An additional factor in optimal migration theory is safety [44]. Although flying high provides a better vantage for landmarks, navigating at low altitudes allows for better assessing ground speed and direction (and thus wind support and drift). Thus, flying at low altitude might reduce navigational uncertainty and minimise the use of energy and time. As a third and final potential explanation of the apparent preference in far eastern curlew and whimbrel to fly at low altitude is that the thinner (low partial oxygen pressure) and generally dry air at high altitude will require increased pulmonary ventilation and promote water loss [11, 45].

### Importance of wind support

After the strong preference for flying at low altitude, wind support was the next most important factor determining the birds’ apparent flight altitude selection. For aerial migrants, wind support can reduce both energy and time cost of migration considerably [46], with wind assistance potentially doubling a bird’s ground speed [3]. A large body of literature shows that avian migrants often time their departures and migrate when they can enjoy wind support (1, 9 and references therein). Many radar studies suggest that migrants predominantly choose flight altitudes at which favourable winds prevail [6, 7, 16]. However, in more recent times there has been some fine-tuning of this general notion, with radar and tracking studies suggesting that migrants may stay at low altitude unless there are headwinds [17, 26]. In selecting flight altitude, migrants may find it difficult to predict wind conditions higher than they are currently flying. Another radar study suggested that migrants may only climb until wind conditions no longer improve irrespective of potentially better wind conditions higher up [18]. However, far eastern curlew and whimbrel seemingly behaved in accordance with this strategy for only 36 and 38% of the time, respectively and this hypothesis was not supported in either of the two species when we investigated the additional effect of actual flight altitude using the conditional logistic mixed effect models. In summary, while our study is consistent with other studies concerning wind support and migration, our study suggests that wind assistance is secondary to a prime tendency to fly at low altitudes regardless of more favourable wind support in higher air layers.

### Impacts of cloud cover

Although avian migrants have been observed flying over and under and even within clouds [9, 21], cloud cover has long been thought to influence animal migration by reducing visibility and hampering navigation, and increasing the chance of encountering precipitation which can cause mortality during migration [47, 48]. Cloud cover and precipitation have been shown to negatively affect migration intensity [12, 49], yet, the effect of cloud cover on flight altitude has rarely been investigated. Irrespective of whether navigating using celestial cues or landmarks, we expected navigation in far eastern curlews and whimbrels during migration to be aided by avoiding clouds. However, only for the former did cloud cover influence flight altitude selection, although, with the limited data available, there was also a tendency for whimbrel to avoid cloud cover. It should also be noted that as both species mostly undertook their migratory flights under clear skies without clouds anywhere in the air column, there was relatively little data available to assess the role of cloud cover in flight altitude selection.

### Impacts of temperature and humidity

High temperature and low humidity can cause water loss due to evaporative cooling [50], which can result in shorter maximum flight ranges [5, 11]. It has been suggested previously that migrants might choose their flight altitude to optimise their water balance and reduce the risk of having to interrupt migration to replenish water stores [11, 19, 20]. However, empirical studies based on radar observations have found mixed support for this hypothesis [6, 19, 20]. Tracking of great reed warblers and black-tailed godwits showed that their high altitude flights were associated with high air temperature at low altitude, suggesting they were potentially avoiding overheating [14, 25] and conserving their water balance. Our finding showed that altitude at which both far eastern curlew and whimbrel chose to fly were neither directly related to air temperature nor relative humidity. However, there was a clear effect of air temperature in combination with altitude suggesting they do not only prefer to fly at low altitude but notably when those low altitudes are cool, which would reduce the risk of overheating. It should also be noted that we never recorded similarly extremely high temperature conditions in this study as Senner et al. [25] did. Moreover, both far eastern curlew and whimbrel may have maintained their water balance by flying at low altitude, which promotes low pulmonary ventilation [11, 45]. Alternatively, as shown in eider ducks (*Somateria mollissima*), migrants might also deal with overheating and water imbalance by shortening migratory flight durations along the route [51], allowing for more frequent recuperation breaks.

### Other potential factors in flight altitude selection

It should be stressed that, despite the strong preference for staying low, birds in this and many other studies [3, 8, 10, 14, 17, 25, 52] occasionally climb to great altitudes. Indeed, our conditional logistic mixed effect modelling evaluating the contribution of the conditions aloft and flight altitude explained 67% and 61% of the variation in far eastern curlew and whimbrel respectively. There are thus other factors determining flight altitude selection in these birds. For example, extremely high temperatures at ground level (not observed in our study) might drive birds to high altitudes [25], as could the rare but regular use of low-level jets (a narrow region of anomalously strong winds that blows in the lower troposphere [10]). Another factor may be whether birds are flying over land or sea. Unfortunately, our analyses did not allow including surface type (i.e. land or sea) as an additional explanatory variable since the conditional logistic models rely on the variations among air layers in the air column where an in-flight fix occurred rather than the variations among air columns/fixes. And it is only among air columns or fixes that surface type varies. However, our data did indicate that both species flew higher over land. We can only speculate on why this is, but variable ground levels and the risk of encountering obstacles while crossing land, but not sea, are obvious candidates in explaining these differences. Irrespective of these differences in flight altitudes over land and over sea, models for both species in which we subsetted the data for fixes above land and above sea showed similar patterns as observed in the main models with the amalgamated data set (additional file Table S3).

### Errors in flight altitude estimation

The recorded negative flight altitudes highlight that realised accuracy in flight altitude estimation at least occasionally far exceeded the nominal accuracy of ±30 m. As explained in the methods this reduces the power of our analyses but need not have a detrimental effect on our conclusions, under the critical assumption that there is no serious bias in the altitudinal measurement errors. We encourage future studies to endeavour increasing sampling frequency of GPS fixes to allow for error correction using state space modelling [30].

## Conclusion

Our study shows that the primary determinant of flight altitude selection is a preference for low altitude. We next find that wind support is an important secondary factor, meaning that the birds in our study not exclusively flew at altitudes at which wind support was best, because staying low has far more advantages for long-distance migrants who are concerned not only with time and energy, but also safety and water balance. As a final caveat it should be considered that the importance of the different factors considered in determining flight altitude need not inform about their overall importance and ranking in shaping migration strategies, since aerial migrants can also take these factors into consideration in when to embark on and stop migratory flights (e.g. [1, 3, 9, 12, 13, 53].

The annual costs of bird strikes to the global commercial aviation industry runs in the billions of dollars. Understanding the drivers of air space use in birds is thus of great importance from both an economic and a conservation perspective [54, 55]. We consider that the here presented quantitative analysis of increasingly available in-flight and atmospheric data, can importantly assist in deepening that understanding, developing risk analyses and potential mitigation strategies. This knowledge is of particular importance in the face of climate change, which not only results in increasing temperatures but also altered wind regimes [56, 57], and patterns of extreme weather events [58, 59], which have already been predicted to endanger migration routes in some species [60].

## Supplementary Information

**Additional file 1: Table S1.** A summary of information on catching sites, deployment dates, number and type of deployed transmitters for both far eastern curlew and whimbrel. **Fig. S1**. The distribution of ground speed measurements acquired by the transmitters for far eastern curlew and whimbrel. **Fig. S2**. Relationships between atmospheric conditions at the actual flight altitude of far eastern curlew and whimbrel. **Table S2**. Atmospheric conditions at the actual flight altitude of far eastern curlew and whimbrel during northbound and southbound migration. **Table S3**. Effects of atmospheric conditions and altitude on far eastern curlew’s and whimbrel’s flight altitude selection as estimated using conditional logistic mixed effect modelling.

## Data Availability

The datasets underlying the analyses are available from Dryad 10.5061/dryad.zpc866t7q [31]. The tracks for far-eastern curlew are available from Movebank (https://www.movebank.org/cms/webapp?gwt_fragment=page=studies,path=study1450838481).
