# Correction to: Far eastern curlew and whimbrel prefer flying low - wind support and good visibility appear only secondary factors in determining migratory flight altitude

**DOI:** 10.1186/s40462-021-00289-z

**Published:** 2021-10-20

**Authors:** Batbayar Galtbalt, Amanda Lilleyman, Jonathan T. Coleman, Chuyu Cheng, Zhijun Ma, Danny I. Rogers, Bradley K. Woodworth, Richard A. Fuller, Stephen T. Garnett, Marcel Klaassen

**Affiliations:** 1 Centre for Integrative Ecology, School of Life and Environmental Science, Deakin University, Geelong, Victoria Australia; 2 Threatened Species Recovery Hub, National Environment Science Program, Research Institute for Environment and Livelihoods, Charles Darwin University, Ellengowan Drive, Casuarina, Northern Territory 0909 Australia; 3 Queensland Wader Study Group, 22 Parker Street, Shailer Park, 4128 Australia; 4 Ministry of Education Key Laboratory for Biodiversity Science and Ecological Engineering, Coastal Ecosystems Research Station of the Yangtze River Estuary, Institute of Biodiversity Science, School of Life Sciences, Fudan University, Shanghai, 200433 China; 5 Department of Environment, Water, Land and Planning, Arthur Rylah Institute, PO Box 137, Heidelberg, Victoria 3084 Australia; 6 Australasian Wader Studies Group, Melbourne, Victoria Australia; 7 School of Biological Sciences, University of Queensland, Brisbane, Queensland Australia; 8 Victorian Wader Study Group, Melbourne, Victoria Australia

## Correction to: *Movement Ecology* (2021) 9:32 https://doi.org/10.1186/s40462-021-00267-5

Following publication of the original article [1], errors were identified in the presentation of some of the reference citations in the Background section (page 2 of the PDF) and the Conclusion section (page 10 of the PDF) due to a typesetting mistake.

These errors have been corrected and the original article has been updated. The publisher apologises to the authors and readers for the inconvenience caused by this mistake.
