# Supplementary material for: Far eastern curlew and whimbrel prefer flying low - wind support and good visibility appear only secondary factors in determining migratory flight altitude
Source: Mov Ecol. 2021 Jun 13;9:32. doi: 10.1186/s40462-021-00267-5 (PMC8201695; doi:10.1186/s40462-021-00267-5)
Supplement: Supplementary file 1 — Additional file 1: Table S1. A summary of information on catching sites, deployment dates, number and type of deployed transmitters for both far eastern curlew and whimbrel. Fig. S1. The distribution of ground speed measurements acquired by the transmitters for far eastern curlew and whimbrel. Fig. S2. Relationships between atmospheric conditions at the actual flight altitude of far eastern curlew and whimbrel. Table S2. Atmospheric conditions at the actual flight altitude of far eastern curlew and whimbrel during northbound and southbound migration. Table S3. Effects of atmospheric conditions and altitude on far eastern curlew’s and whimbrel’s flight altitude selection as estimated using conditional logistic mixed effect modelling. [file 40462_2021_267_MOESM1_ESM.docx]

*Additional file*

FAR EASTERN CURLEW AND WHIMBREL PREFER FLYING LOW - WIND SUPPORT AND GOOD VISIBILITY APPEAR ONLY SECONDARY FACTORS IN DETERMINING MIGRATORY FLIGHT ALTITUDE

Table S1. A summary of information on catching sites, deployment dates, number and type of deployed transmitters for both far eastern curlew and whimbrel.

| Species | Site | Date | Individuals | Tag type | Tag weight (g) |
| --- | --- | --- | --- | --- | --- |
|  |  |  |  |  |  |
| Far eastern curlew | Roebuck Bay, Broome in NW Australia | 18 and 21/02/2019 | 8 | Ornitela GPS-GSM | 15 |
|  | Darwin Harbour, Australia | 21/11/2017, 9/11/2018 | 3 | Ornitela GPS-GSM | 20 |
|  | Yellock Creek, Western Port Victoria | 13/01/2019 | 3 | Ornitela GPS-GSM | 15 |
|  | Moreton Bay, Queensland, Australia | 03/03/2018, 13/01/2019 | 3 | Ornitela GPS-GSM | 15 |
| Whimbrel | Roebuck Bay, Broome in NW Australia | 24/02/2018 | 9 | GPS-GSM | 7 |


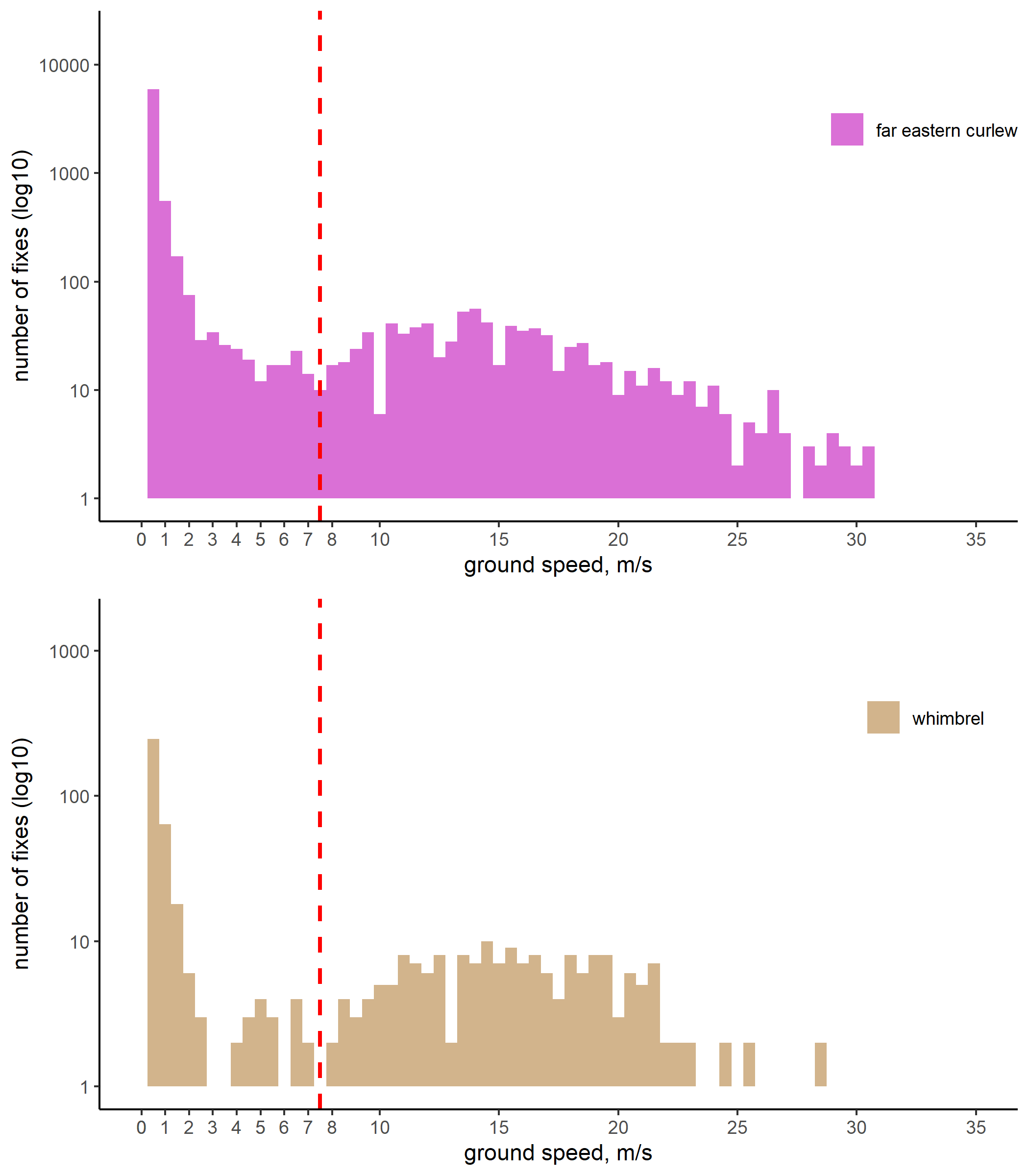


Figure S1. The distribution of ground speed measurements acquired by the transmitters for far eastern curlew (top panel) and whimbrel (bottom panel). Red line indicates the cut-off value of 7.5 m/s used for distinguishing between in-flight and stationary points. Ground speed measurements are binned by 0.5 m/s. The number of fixes in each bin are log transformed for plotting purposes.


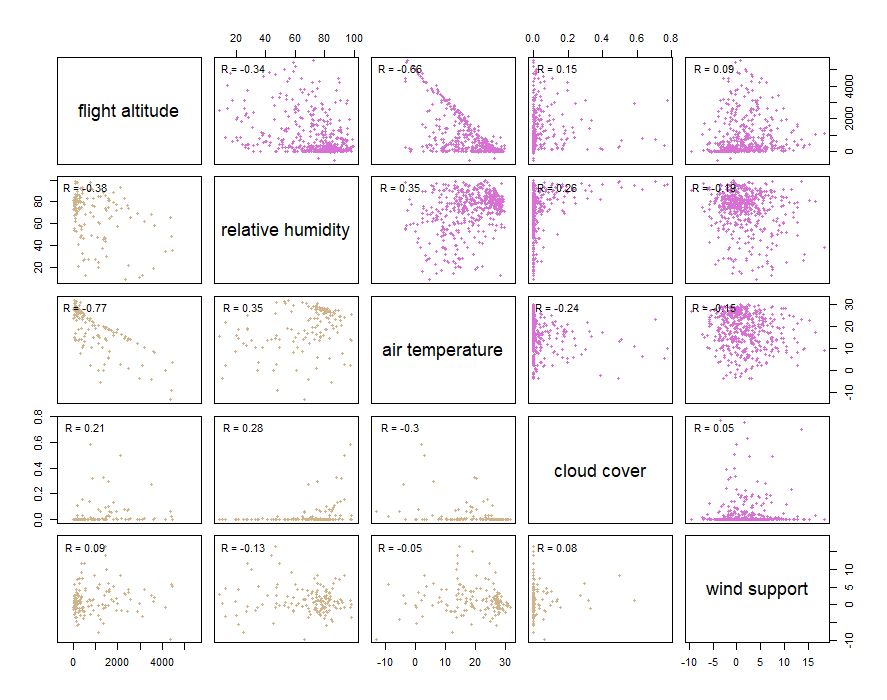


Figure S2. Relationships between atmospheric conditions at the actual flight altitude of far eastern curlew (purple) and whimbrel (brown) data. Pairwise correlation coefficients are provided for each comparison.

Table S2. Atmospheric conditions at the actual flight altitude of far eastern curlew and whimbrel during northbound and southbound migration. Mann-Withney-Wilcoxon tests were used to examine if two samples differed significantly. P<0.005 are shown in bold.

| Variables | Migratory direction |  | far eastern curlew | | |  | whimbrel | | | Wilcox. |
| --- | --- | --- | --- | --- | --- | --- | --- | --- | --- | --- |
|  |  |  | Mean (range) | SD | N |  | Mean (range) | SD | N |  |
|  |  |  |  |  |  |  |  |  |  |  |
| Wind support, m/s | Northbound |  | 1.26 (-9.65 – 18.4) | 4.1 | 320 |  | 1.26 (-9.7 – 16.7) | 4.1 | 129 | p=0.96 |
|  | Southbound |  | 1.96 (-7.34 – 15.3) | 4.4 | 192 |  | 1.35 (-4.76 – 5.9) | 3.1 | 17 | p=0.85 |
|  |  | | p=0.11 | | |  | p=0.56 | | |  |
|  |  |  |  |  |  |  |  |  |  |  |
| Cloud cover | Northbound |  | 0.03 (0 – 0.77) | 0.1 | 320 |  | 0.08 (0 – 0.6) | 0.1 | 129 | **p<0.05** |
|  | Southbound |  | 0.03 (0 – 0.76) | 0.1 | 192 |  | 0.06 (0 – 0.3) | 0.1 | 17 | p=0.77 |
|  |  | | p=0.92 | | |  | p=0.58 | | |  |
|  |  |  |  |  |  |  |  |  |  |  |
| Temperature, °C | Northbound |  | 17.9 (-3.3 – 29.1) | 8.0 | 320 |  | 20.3 (-13.1 – 31.7) | 9.0 | 129 | **p<0.001** |
|  | Southbound |  | 19.2 (-3.4 – 29.8) | 7.8 | 192 |  | 13.8 (-9.2 – 27.8) | 10.5 | 17 | **p<0.05** |
|  |  | | p=0.11 | | |  | **P<0.01** | | |  |
|  |  |  |  |  |  |  |  |  |  |  |
| Relative humidity, % | Northbound |  | 71.9 (13 – 98.8) | 19.7 | 320 |  | 69.0 (11 – 98.3) | 20.0 | 129 | p=0.07 |
|  | Southbound |  | 73.7 (8.8 – 99.4) | 16.4 | 192 |  | 61.4 (8.81 – 90.8) | 24.7 | 17 | **p<0.05** |
|  |  | | p=0.90 | | |  | p=0.19 | | |  |

Table S3. Effects of atmospheric conditions and altitude on far eastern curlew’s and whimbrel’s flight altitude selection as estimated using conditional logistic mixed effect modelling. All explanatory variables are scaled. Interaction of altitude and air temperature is included to account for collinearity. Coefficients of variables, standard errors (SE), 95% confidence interval (CIs), odds ratios and associated p-values of variables are presented. P<0.05 are shown in bold. Sample size (n) and goodness of fit (R^2^) of each models are presented in bracket.

|  | Coefficient (±SE) | 95% CI | Odds ratio | Z | p | Random effect | |
| --- | --- | --- | --- | --- | --- | --- | --- |
|  |  |  |  |  |  |  |  |
| **Far eastern curlew – over land (n=146 [1,760], R^2^=0.63)** | | |  |  |  | σ^2^ | Std. dev. |
| Altitude | -2.34 (0.87) | -4.03/-0.64 | 0.10 | -2.70 | **<0.01** |  |  |
| Wind support | 0.90 (0.26) | 0.39/1.41 | 2.46 | 3.47 | **<0.001** |  |  |
| Cloud cover | -0.44 (0.20) | -0.84/-0.04 | 0.64 | -2.17 | **<0.05** |  |  |
| Relative humidity | 0.64 (0.36) | -0.07/1.35 | 1.90 | 1.77 | 0.08 |  |  |
| Air temperature | -0.68 (0.95) | -2.54/1.18 | 0.51 | -0.71 | 0.48 |  |  |
| Altitude : Air temperature | -0.04 (0.18) | -0.39/0.30 | 0.96 | -0.24 | 0.81 |  |  |
| Individual | - | - | - | - | **-** | 0.02 | 0.0004 |
|  | | |  |  |  |  |  |
| **Far eastern curlew – over sea (n=335 [4,367], R^2^=0.65)** | | |  |  |  |  |  |
| Altitude | -2.42 (0.53) | -3.45/-1.38 | 0.09 | -4.57 | **<0.001** |  |  |
| Wind support | 0.87 (0.18) | 0.51/1.22 | 2.38 | 4.80 | **<0.001** |  |  |
| Cloud cover | -0.36 (0.11) | -0.58/-0.14 | 0.70 | -3.15 | **<0.01** |  |  |
| Relative humidity | 0.05 (0.19) | -0.33/0.43 | 1.05 | 0.25 | 0.80 |  |  |
| Air temperature | -0.87 (0.52) | -1.88/0.15 | 0.42 | -1.67 | 0.09 |  |  |
| Altitude : Air temperature | -0.31 (0.09) | -0.49/-0.14 | 0.73 | -3.52 | **<0.001** |  |  |
| Individual | - | - | - | - | **-** | 0.02 | 0.0004 |
|  |  |  |  |  |  |  |  |
| **Whimbrel – over land (n=57 [675], R^2^=0.55)** | | |  |  |  |  |  |
| Altitude | -1.82 (1.31) | -4.40/0.75 | 0.16 | -1.39 | 0.17 |  |  |
| Wind support | 0.48 (0.44) | -0.39/1.34 | 1.61 | 1.08 | 0.28 |  |  |
| Cloud cover | -0.35 (0.41) | -1.15/0.44 | 0.70 | -0.87 | 0.39 |  |  |
| Relative humidity | -0.30 (0.50) | -1.28/0.67 | 0.74 | -0.60 | 0.55 |  |  |
| Air temperature | -0.32 (1.48) | -3.21/2.57 | 0.73 | -0.22 | 0.83 |  |  |
| Altitude : Air temperature | -0.19 (0.20) | -0.58/0.20 | 0.83 | -0.95 | 0.34 |  |  |
| Individual | - | - | - | - | **-** | 0.02 | 0.0004 |
|  |  |  |  |  |  |  |  |
| **Whimbrel – over sea (n=89 [1,157], R^2^=0.58)** | | |  |  |  |  |  |
| Altitude | -3.05 (1.30) | -5.60/-0.50 | 0.05 | -2.34 | **<0.05** |  |  |
| Wind support | 0.81 (0.32) | 0.19/1.42 | 2.24 | 2.55 | **<0.05** |  |  |
| Cloud cover | 0.32 (0.24) | -0.14/0.79 | 1.38 | 1.35 | 0.18 |  |  |
| Relative humidity | -0.70 (0.35) | -1.38/-0.01 | 0.50 | -1.99 | **<0.05** |  |  |
| Air temperature | -0.51 (1.30) | -3.06/2.04 | 0.60 | -0.39 | 0.70 |  |  |
| Altitude : Air temperature | -0.37 (0.27) | -0.90/0.16 | 0.69 | -1.38 | 0.17 |  |  |
| Individual | - | - | - | - | **-** | 0.02 | 0.0004 |
|  |  |  |  |  |  |  |  |
